# Do Increased Doses to Stem-Cell Niches during Radiation Therapy Improve Glioblastoma Survival?

**DOI:** 10.1155/2016/8793462

**Published:** 2016-06-27

**Authors:** Sebastian Adeberg, Semi Ben Harrabi, Nina Bougatf, Denise Bernhardt, Angela Mohr, Juliane Rieber, Christian Koelsche, Stefan Rieken, Juergen Debus

**Affiliations:** ^1^Department of Radiation Oncology, University Hospital Heidelberg, Im Neuenheimer Feld 400, 69120 Heidelberg, Germany; ^2^Clinical Cooperation Unit Radiation Oncology, German Cancer Research Center (DKFZ), Im Neuenheimer Feld 280, 69120 Heidelberg, Germany; ^3^Heidelberg Ion-Beam Therapy Center (HIT), Im Neuenheimer Feld 450, 69120 Heidelberg, Germany; ^4^Heidelberg Institute of Radiation Oncology (HIRO), University Hospital Heidelberg, Im Neuenheimer Feld 400, 69120 Heidelberg, Germany; ^5^Department of Medical Physics in Radiation Oncology, German Cancer Research Center (DKFZ), Im Neuenheimer Feld 280, 69120 Heidelberg, Germany; ^6^Department of Neuropathology, University Hospital Heidelberg, Im Neuenheimer Feld 224, 69120 Heidelberg, Germany; ^7^German Cancer Consortium (DKTK), Clinical Cooperation Unit Neuropathology, German Cancer Research Center (DKFZ), 69120 Heidelberg, Germany

## Abstract

*Background and Purpose*. The reasons for the inevitable glioblastoma recurrence are yet understood. However, recent data suggest that tumor cancer stem cells (CSCs) in the stem-cell niches, with self-renewing capacities, might be responsible for tumor initiation, propagation, and recurrence. We aimed to analyze the effect of higher radiation doses to the stem-cell niches on progression-free survival (PFS) and overall survival (OS) in glioblastoma patients.* Materials and Methods*. Sixty-five patients with primary glioblastoma treated with radiation therapy were included in this retrospective analysis. The SVZ and DG were segmented on treatment planning magnetic resonance imaging, and the dose distributions to the structures were calculated. The relationship of dosimetry data and survival was evaluated using the Cox regression analysis.* Results*. Conventionally fractionated patients (*n* = 54) who received higher doses (*D*_mean_ ≥ 40 Gy) to the IL SVZ showed improved PFS (8.5 versus 5.2 months; *p* = 0.013). Furthermore, higher doses (*D*_mean_ ≥ 30 Gy) to the CL SVZ were associated with increased PFS (10.1 versus 6.9 months; *p* = 0.025).* Conclusion*. Moderate higher IL SVZ doses (≥40 Gy) and CL SVZ doses (≥30 Gy) are associated with improved PFS. Higher doses to the DG, the second stem-cell niche, did not influence the survival. Targeting the potential cancer stem cells in the SVZ might be a promising treatment approach for glioblastoma and should be addressed in a prospective randomized trial.

## 1. Introduction

The heterogeneity observed in glioblastoma carcinogenesis has been described by a stochastic model [1]. However, there is evidence that glioblastoma progression and initiation are attributed to glioma stem cells. Glioblastoma may be organized hierarchically [2], with a subset of stem cells responsible for self-renewing properties, the ability to migrate, tumor initiation and progression, and multilineage potency [3–6]. It is yet unknown whether these stem cells are the originators of primary central nervous system malignancies, but they may play a major role in the response to antitumor treatments.

In the human brain, two anatomical regions have been shown to harbor neuronal stem cells (NSCs): first, the subventricular zone (SVZ), a 3–5 mm thick region which is located adjacent to the lateral ventricles, and second, the subgranular layer of the dentate gyrus (DG), which is a subsection of the hippocampal formation [7, 8]. These NSCs in the stem-cell niches maintain the ability for neurogenesis throughout adulthood [9, 10]. The SVZ is hypothesized to also harbor cancer stem cells (CSCs) [11]. Furthermore, NSCs are suspected to dedifferentiate into CSCs through a series of oncogene and tumor suppressor gene mutations. Thus, glioblastomas infiltrating the SVZ are associated with decreased survival [12] and a higher rate of multifocal and distant recurrences [13].

Following the CSC hypothesis, all CSCs have to be devitalized to eliminate the tumor. However, current therapy strategies do not target NSCs or CSCs in the stem-cells niches. Chemo- and radiation therapy resistance of CSCs and suspected lack of penetration of chemotherapy into the stem-cell niches may explain the failure of the present therapy regimens [14, 15]. Furthermore, the cerebral stem-cell niches are not intentionally included in the target volume during radiation therapy.

Increased doses to the ipsilateral (IL) [16–19] and bilateral SVZ [20] have been shown to improve outcome in glioblastoma patients. We therefore aim to assess the influence of dose distributions on the SVZ and DG during postoperative radiation therapy in our institution.

## 2. Materials and Methods

65 patients that received radiation therapy at the Department of Radiation Oncology, University Hospital Heidelberg, between May 2010 and December 2012 were retrospectively identified. Selection criteria included age over 18 years, histopathologically proven primary supratentorial glioblastoma, sufficient MR imaging with preoperative and initial postoperative imaging (see Follow-Up), available radiotherapy treatment planning CT dataset on the Oncentra MasterPlan® (Elekta®, Stockholm, Sweden) planning system, and documented progression or death. Only patients who completed the treatment plan were included in the analysis. The Karnofsky performance status (KPS) was assessed before treatment initiation. All patients received a 3D conformal radiation therapy (3D-CRT) plan with a median dose of 60.0 Gy (range: 40.05–68 Gy) in a median dose of 2.0 Gy per fraction (range: 1.8–2.67 Gy) prescribed on the PTV. Only one patient received dose escalation up to 68 Gy on a rather small target volume. Hypofractionated radiation therapy was mainly performed in elderly patients. Concurrent and adjuvant temozolomide therapy was applied (with at least two completed cycles). Gross total resection (complete resection of the preoperative contrast enhancement) or subtotal resection (residual contrast enhancement) was defined based on postoperative MR imaging and reviewed by an experienced radiologist. MGMT promoter methylation status determination was carried out as previously described [21]. The study was approved by the local ethics committee (number S-056/2015).

### 2.1. Contouring and Treatment Planning

Contouring was performed on patient's original treatment planning computed tomography (CT) scans, coregistered with postoperative magnet resonance imaging using contrast-enhanced T1-weighted sequences. The initial gross tumor volume (GTV) was defined as the contrast-enhancing lesion on T1-weighted sequences and hyperintense low-grade tumor mass, surgical resection cavity, and perifocal edema on T2 fluid-attenuated inversion recovery (FLAIR). The clinical target volume (CTV) included an added 2-3 cm margin accounting for microscopic spread. To account for technical inaccuracies, a safety margin of 3 mm was added for the planning treatment volume (PTV).

**Figure 1:**
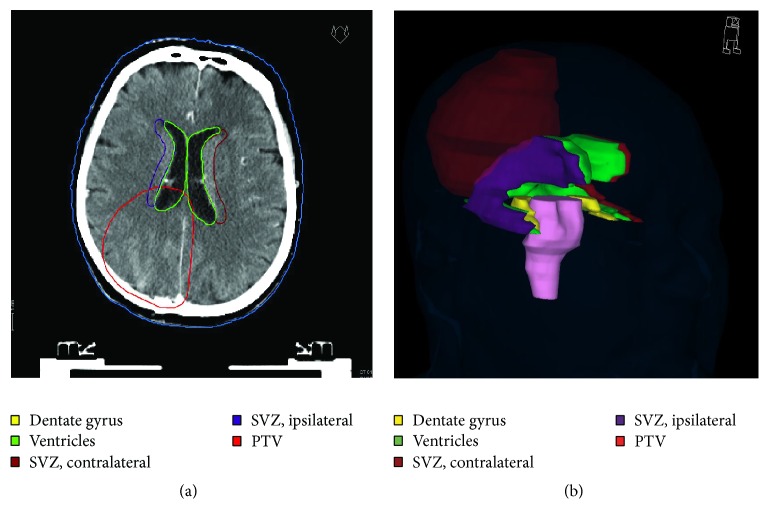
(a) Axial planning computed tomography with ventricles, subventricular zone (SVZ), and PTV. (b) 3D reconstruction including structures shown in the legend. The SVZ is defined as a 5 mm margin lateral to the lateral ventricle (purple and dark red). The brainstem (violet) is included for anatomical orienting.

IL and contralateral (CL) ventricles were contoured using coregistered postoperative MR and CT imaging. To allocate the laterality, we determined the tumor key area, regardless of bilateral tumor growth. IL and CL SVZ were contoured as a 5 mm margin lateral to the lateral ventricles (Figure 1) [20, 22]. The IL and CL DG, as a part of the hippocampal formation, were defined in accordance with previous published guidelines [23] and the RTOG contouring atlas.

Replanning was performed on original planning CT datasets and dose recalculation was done using the initial planning parameters. Usually, organs at risk (OAR) include the brainstem, the optic nerves, chiasm, eyes, lenses, and spinal cord. Depending on treatment volume localization additional structures need to be considered. Organs at risk (OAR) were considered like in the previous treatment plan. Dose volume histograms (DVHs) were constructed for all volumes and *D*_mean_ values were extracted. Conventionally fractionated patient's OAR doses were stratified by a dose of ≥40 Gy and <40 Gy. The cutoff value for the CL SVZ and DG was set as ≥30 Gy and <30 Gy due to the low mean doses. The cutoff values were determined escalating and deescalating the dose values. To include patients with hypofractionated radiation therapy in the analysis, doses to OAR were calculated as biological effective doses, taken as *α*/*β* of 2.0 for normal brain tissue. However, the numbers of hypofractionated patients were too small to generate a valid statistical analysis in this subgroup and further analyses were carried out only for conventionally fractionated patients.

### 2.2. Data Management and Automatic Dose Volume Analysis

All data was retrospectively imported in a central research database acting as central data source [24]. Dose volume analysis has been performed in a central analysis platform. Workflow has been designed to analyze the original radiotherapeutic imaging data (RT data) of all patients with the abovementioned contouring automatically. First, RT data has been retrieved from the central research database and it has been preprocessed for analysis. During analysis, dose statistics and dose volume histograms (DVHs) have been calculated automatically. All results have been written into central storage of the analysis platform. Finally, results of all patients were summarized in one single result file for further statistical analysis.

### 2.3. Follow-Up

Patient data including MR images were assessed before therapy, 6 weeks after radiation therapy, and at 3-month intervals until progression/recurrence or death. Pretherapeutic tumor localization and posttherapeutic tumor progression were determined by a radiology specialist according to the RANO criteria [25]. Minimum follow-up interval in our patient cohort was 12 months (range: 12–54 months). Sixteen patients (24.6%) were still alive at the time of analysis. Progression-free survival (PFS) was calculated from the day of commencement of radiation therapy till the occurrence of progression based on contrast-enhanced T1-weighted MR imaging on axial and coronal images. The time between the day of the first diagnosis and the day of death was valued as the overall survival (OS). If the physician suspected pseudoprogression, further follow-up MRI was made to clarify true radiographic progression. All survival data were censored if death without diagnosis of progression or without follow-up examination occurred.

### 2.4. Statistical Analysis

Statistical analysis was carried out using SigmaPlot*™* (Systat Software GmbH, Germany). Survival rates were calculated using the Kaplan-Meier method with a 95% confidence interval. Survival rates were compared using the log-rank test. Univariate (for covariates, see Tables 3 and 4) and multivariate Cox regression analyses were performed to compare survival rates in regard of covariates (KPS, tumor localization, MGMT promoter status, surgical resection status, temozolomide therapy, *D*_mean_ IL SVZ ≥ 40 Gy, and *D*_mean_ CL SVZ ≥ 30 Gy).

## 3. Results

Patients undergoing radiation therapy at the Department of Radiation Oncology, University Hospital Heidelberg, between May 2010 and December 2012 were screened via an institute's database search. 65 patients matched the studies inclusion criteria. Patients' characteristics of the whole cohort and subgroups with conventionally fractionated (*n* = 54) and hypofractionated (*n* = 11) radiation therapy are depicted in Table 1. It is shown that the subgroups are reasonably balanced. All patients completed the prescribed fractionation protocol. 46 patients received concomitant temozolomide therapy according to the Stupp protocol [26]. Tumor localization was divided into centrally located and peripherally located tumors like previously described [13]. In 39 (72.2%) of the conventionally fractionated cases, the tumor was localized within <10 mm to the ventricle system (median: 3 mm) and in 15 cases (27.8%) ≥ 10 mm distant from the ventricle system (median: 23 mm). Salvage therapy at recurrence was decided by the treating physician and covered a large spectrum, amongst reirradiation (5/65, 7.7%), reresection (5/65, 7.7%), and systemic therapy (35/65, 53.9%).

The mean volume of the IL ventricle was 21.39 mL (9.33–44.50 mL), of the CL ventricle was 23.14 mL (2.95–23.14 mL), of the IL SVZ was 14.05 mL (8.41–22.80 mL), of the CL SVZ was 14.50 mL (8.68–23.80 mL), of the IL DG was 3.35 mL (1.27–6.60 mL), and of the CL DG was 3.43 mL (1.06–6.98 mL).

Mean PTV volume was 342.0 mL (117.4–674.7 mL). In conventionally fractionated patients, mean PTV volume was 280.6 mL (200.9–530.9 mL) and mean PTV volume of hypofractionated patients was 351.0 mL (117.4–674.7).

*D*_mean_ to the IL ventricle was 39.77 Gy (14.51–56.37 Gy), to the CL ventricle was 25.28 Gy (5.29–46.68 Gy), to the IL SVZ was 40.67 Gy (14.84–56.87 Gy), to the CL SVZ was 20.86 Gy (4.10–45.07 Gy), to the IL DG was 33.30 Gy (3.32–55.95 Gy), and to the CL DG was 13.15 Gy (1.47–52.32 Gy).

**Figure 2:**
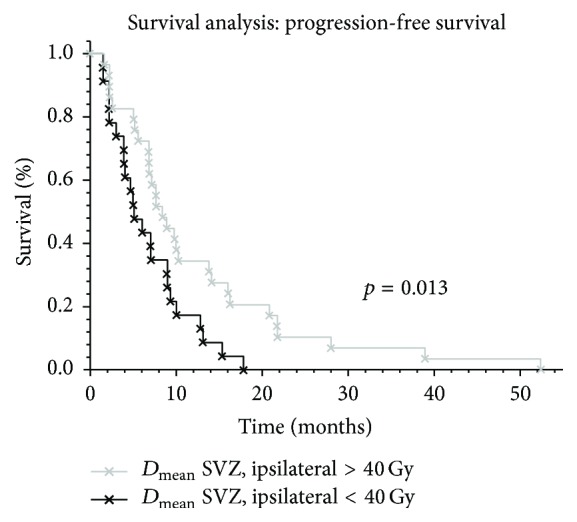
Influence of increased doses (*D*_mean_ > 40 Gy) to the ipsilateral subventricular zone (SVZ) in glioblastoma patients.

**Figure 3:**
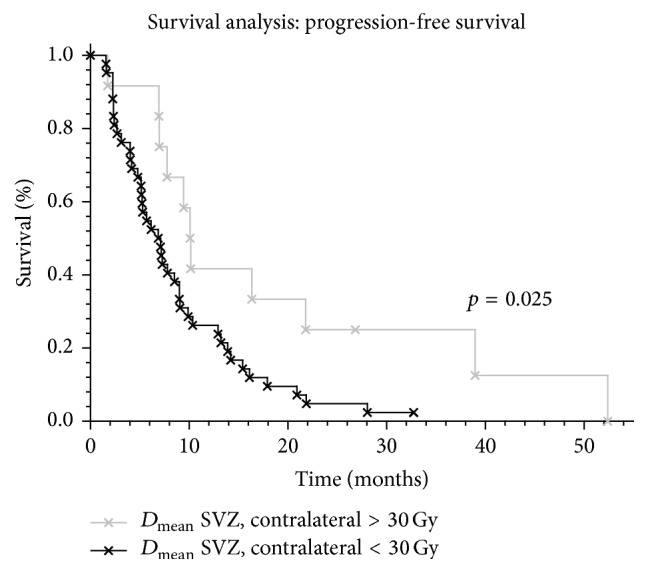
Influence of increased doses (*D*_mean_ > 30 Gy) to the contralateral subventricular zone (SVZ) in glioblastoma patients.

Median PFS of the study group was 7.1 months (1.6–52.4 months) and median OS was 20.8 months (4.3–53.8 months). Median PFS rates (4.3 versus 7.8 months; *p* = 0.18) and median OS rates (17.0 versus 21.3 months; *p* = 0.32) of hypofractionated patients were not significantly inferior compared to conventionally fractionated patients, respectively. Conventionally fractionated patients who received higher doses to the IL SVZ (*D*_mean_ ≥ 40 Gy) showed increased PFS compared to patients with lower doses (8.5 versus 5.2 months; *p* = 0.013) with HR of 0.40 (95% CI: 0.24–0.78; *p* = 0.002) (Figure 2 and Tables 2 and 3). Similar findings could be observed in this group for higher doses (*D*_mean_ ≥ 30 Gy) to the CL SVZ (10.1 versus 6.9 months; *p* = 0.025) (Figure 3 and Tables 2 and 3). Peripheral tumor localization was not associated with increased PFS (*p* = 0.55). However, OS showed a trend towards improved survival in this patient subgroup (*p* = 0.073). Interestingly, the ratio of patients receiving > 40 Gy on the IL SVZ was similar in regard of the tumor localization to the SVZ (central 64.0% versus peripheral 55.6%; *p* = 0.67).

Surgical tumor resection showed a trend towards improved PFS without reaching statistical significance (HR: 0.48; 95% CI: 0.22–1.03; *p* = 0.06). However, no effects of dose volume relations on OS could be detected (Table 4). The only factor significant in the univariate analysis for OS was temozolomide therapy (HR: 0.49; 95% CI: 0.27–0.90; *p* = 0.02). Eleven hypofractionated patients (16.9%) were included in the study cohort. As expected, no statistically significant effect of dose to OAR could be found in the survival analysis and Cox regression model (data not illustrated).

In the univariate analysis of patients with conventionally fractionated radiation therapy, *D*_mean_ IL SVZ ≥ 40 Gy, *D*_mean_ IL ventricle ≥ 40 Gy, and *D*_mean_ CL SVZ ≥ 30 Gy were significantly associated with PFS (Table 5). In the multivariate model that included KPS, tumor localization, MGMT promoter status, surgical resection status, temozolomide therapy, *D*_mean_ IL SVZ ≥ 40 Gy, and *D*_mean_ CL SVZ ≥ 30 Gy, average CL SVZ dose higher than 30 Gy remained a prognostic factor for PFS. For OS, temozolomide therapy remained the only predictor (Table 5).

To assess whether 40 Gy to the IL SVZ and 30 Gy to the CL SVZ were the minimal threshold values for increased PFS in conventionally fractionated patients, further survival and Cox regression survival analyses were performed. We analyzed 30 Gy (*n* = 53) and 50 Gy (*n* = 16) for the IL SVZ and 20 Gy (*n* = 30) and 40 Gy (*n* = 2) for the CL SVZ. *D*_mean_ of more than 30 Gy showed a tendency towards improved PFS (HR: 0.56; 95% CI: 0.30–1.08; *p* = 0.08). Altogether, no significant improvement of the OS could be found in association with the abovementioned mean OAR doses.

## 4. Discussion

In this study, we show that increased dose to the subventricular zone improves the progression-free survival in glioblastoma patients. We compared patients with different fractionation schemes; however, a dose-response relationship could only be shown in conventional fractionated patients. Given that the standard treatment dose to the PTV is mainly 60.0 Gy and the SVZ was not targeted intentionally, 31 patients received doses ≥40 Gy to the IL SVZ and 12 patients received ≥30 Gy to the CL SVZ. Higher or smaller mean threshold doses to OAR were not detected. *D*_mean_ to the DG did not influence the survival rates.

Current trimodal treatment regimens with maximal safe surgical resection followed by chemoradiation [26] only achieve poor survival rates of approximately 15 months. The majority of patients experience local recurrences [13]. However, distant brain relapses are no exception [13, 27], potentially with an increasing incidence through improved imaging modalities and prolonged survival with new salvage therapy agents. A possible explanation for persistent treatment failure in glioblastoma might be the ability of glioma cells to migrate substantially along cortical fibers [28, 29] into healthy cerebral areas and out of the treatment volume. A controversial discussed hypothesis, the tumor stem-cell hypothesis, suggests that glioblastoma may originate from glioma stem cells and are repopulated by those. Therefore, the SVZ lateral to the lateral ventricle and the DG, two brain stem-cell niches, maintain neurogenic capacities throughout adulthood [9, 10]. The SVZ might represent a potential retreat for CSCs and it is hypothesized that glioma cells are able to recruit neuronal stem cells and induce malignant transformation [30], contributing to glioma propagation, therapy resistance, and recurrence with their self-renewing capacities and the ability to repopulate a tumor [2, 4].

An earlier study [20] investigated improved progression-free survival in 55 high-grade glioma patients if the bilateral SVZ dose was greater than 43 Gy (15.0 versus 7.2 months; *p* = 0.028) with a hazard ratio of 0.73 (*p* = 0.019). The authors concluded that additionally targeting the cerebral stem-cell niches might be superior to targeting the tumor mass alone.

Gupta et al. [17] investigated a small cohort of 40 newly diagnosed glioblastoma patients receiving postoperative conventionally fractionated chemoradiation. Here, increasing *D*_mean_ to the IL SVZ predicted improved OS. However, higher doses than 57.9 Gy to the CL SVZ were associated with decreased PFS and OS. In our cohort, we could show improved PFS if the CL SVZ received doses ≥ 40 Gy. One fundamental difference lies in the contoured SVZ volume. We include the anterior, superior, and inferior aspects of the SVZ with a mean IL volume of 14.05 mL and CL volume of 14.50 mL compared to Gupta who focused particularly on the anterior aspects of the SVZ. Furthermore, excessive dose escalation of the CL SVZ came along with high bilateral doses, which may cause more potentially life-limiting side effects.

In a larger series of 116 glioblastoma patients [16] with postoperative intensity modulated chemoradiation, patients with IL SVZ dose of ≥40 Gy after gross total resection (GTR) had significantly improved PFS compared to lower SVZ doses (15.1 versus 10.3 months; *p* = 0.028; HR: 0.39), respectively. This improvement in the subgroup after GTR could be transferred into improved OS (17.5 versus 15.6 months; *p* = 0.027; HR: 0.39). Restriction of the benefit to the patients who underwent GTR may be explained by the substantial influence of residual tumor on tumor recurrence, which might surpass the influence of SVZ dose on tumor recurrence. Furthermore, the author could not find a correlation with higher CL SVZ doses and decreased patients KPS after radiation therapy which is contradictory with the hypothesis of Gupta et al. and supports our findings. Here, moderate increased doses (≥30 Gy) to the CL SVZ in our cohort improved the PFS (10.1 versus 6.9 months; *p* = 0.025) and might affect the SVZ cells without nullifying this effect by increased side effects.

In contrast to our findings, Slotman et al. [31] could not find a correlation between increased dose to the SVZ (IL: 48.7 Gy, CL: 29.4 Gy, and BL: 37.5 Gy) of 40 primary glioblastoma patients and PFS or OS. Furthermore, lower CL SVZ was associated with a higher incidence of distant cerebral recurrence and no distant recurrence was seen in the subgroup of patients after GTR and CL SVZ doses during radiation therapy ≥43 Gy, without reaching statistical significance. The authors conclude that 43 Gy might be insufficient to neutralize radiation-resistant glioma stem cells in the SVZ [15, 32]. Interestingly, Iuchi et al. reported improved outcomes in patients undergoing hypofractionated radiation therapy that developed necrosis within the SVZ [33].

Glioblastoma growth patterns are hardly understood. However, most glioblastomas are located peripherally [13]. Hence, during disease progression, the majority of glioblastomas become in spatial relation with the ventricle system regardless of the tumor origination [34]. Kappadakunnel and colleagues [35] recognized a relationship between glioblastoma patient's survival rates and stem-cell gene expression, even though no specific glioblastoma stem-cell gene signature could be evidenced.

If the stem-cell hypothesis is true, repopulation in the stem-cell niches and glioblastoma cell migration pattern in and out of the SVZ might be the plausible mechanisms of glioblastoma recurrence and radioresistance.

Our data show that application of higher doses to the IL and CL SVZ improved the progression-free interval. Logically, further improvement of PFS might translate into increased OS, but our study might be underpowered to prove this effect. However, historic data from whole brain radiotherapy (WBRT) with SVZ coverage did not show an advantage in glioblastoma [36–38]. Here, high doses to the entire brain might lead to higher toxicity and obliterate the beneficial effect of SVZ dose and cannot directly be compared to our findings.

The role of dose to the contralateral SVZ in glioblastoma is not clear yet either. Maybe dose to the contralateral SVZ stimulates migration of reparative NSCs whereas in other cases the SVZ in general plays a minor role if the tumor burden is great [39]. Multiple factors that were not evaluated might contribute to our findings. It is possible that the centrally located tumors present earlier with clinical symptoms than their peripheral counterparts. Radiation of the SVZ might induce immunomodulating processes, as CSCs in vitro have been shown to induce regulatory T-cells while inhibiting cytotoxic T-cell activation combined with induction of cytotoxic T-cell apoptosis [40]. The micromilieu of the stem-cell niches, which seems to be a multimodal key regulator for stem-cell behavior [41], is very likely to be influenced in its function by ionizing radiation. Another influence factor could be inflammatory processes induced by radiation therapy, which contribute to depopulation of irradiated regions and impair neurogenesis [42].

The current study has the shortcomings of a retrospective study of nonconsecutive treated patients. We investigated a slightly heterogeneous patient cohort with different treatment regimens and the majority of patients received different salvage therapy which might represent a bias in the survival analysis. MGMT promoter statuses and other prognostic and predictive molecular markers were only available for a subset of patients which could be considered a confounding factor with influence on the survival rates. The rather small number of patients might be a potential bias and therefore the results of the multivariate analysis should be interpreted cautiously. No firm conclusion can be drawn concerning the relation between dosimetry and survival in hypofractionated radiation therapy due to the low patient number, so this merits further investigation. Two clinical trials are being conducted which evaluate higher doses to the stem-cell niches (ClinicalTrials.gov Identifiers: NCT02177578 and NCT02039778) and sparing dose to the neuronal stem cells. Both studies will help to shed light on the ongoing discussion of the benefit of SVZ irradiation.

The strength of this study is the long-term follow-up of the patients. All OAR contours were defined by a single radiation oncologist to minimize intraobserver variability and ensure consistency. The known clinical predictive factors were considered in the Cox regression analysis. Twelve and 31 patients receiving ≥30 Gy to the CL SVZ and ≥40 Gy to the IL SVZ can be considered as a substantial strength of this study, compared to the studies found in the literature, respectively.

It is not clear yet whether and which glioblastoma patients profit from radiation therapy including the stem-cell niche, but there are data supporting the thesis that a subset of glioblastoma occurs with a more aggressive growth pattern and a higher incidence of multifocal occurrence. This subset might be susceptible to intentional SVZ targeting during radiotherapy. This approach could offer a valuable improvement of glioma therapy. In a next step, we addressed this topic, by determining mutational profiles and genome-wide copy number profiles of glioblastoma in regard of the SVZ. These findings might deliver a deeper insight into glioblastoma genetics and serve as a foundation for further dose-response studies in regard of the SVZ. Furthermore, the dose threshold has to be clarified through future studies; likewise, excessive dose escalation to the stem-cell niche [17] might diminish the benefits of the SVZ irradiation and lead to contrary results.

In summary, this study could find an association between moderate higher IL (≥40 Gy) and CL (≥30 Gy) SVZ doses and improved PFS. Higher doses to the DG, the second stem-cell niche, did not influence the survival. OS rates were not associated with dose volume parameters. Even though our retrospective data have to be interpreted cautiously, the approach to target the CSC in the SVZ is promising and should be addressed in a prospective randomized trial.

## Figures and Tables

**Table 1:** Patient characteristics. Numbers in brackets represent percentages and refer to the absolute values in front.

Cofactors	All *n* = 65	Conventionally fractionated patients *n* = 54	Average IL SVZ dose ≥ 40 Gy *n* = 31	Average IL SVZ dose < 40 Gy *n* = 23	Average CL SVZ dose ≥ 30 Gy *n* = 12	Average CL SVZ dose < 30 Gy *n* = 42
Gender						
Male	43 (66.2)	38 (70.4)	21 (67.7)	17 (73.9)	8 (66.7)	34 (81.0)
Female	22 (33.8)	16 (29.4)	10 (32.3)	6 (26.1)	4 (33.3)	8 (19.0)

Median age in years (range)	58.9 (29.5–78.3)	55.1 (29.5–78.3)	53.8 (39.7–75.5)	59.1 (29.5–77.8)	53.6 (40.8–73.4)	56.2 (29.5–77.8)
Median Karnofsky performance status in % (range)	90 (60–100)	90 (70–100)	90 (70–100)	90 (70–100)	90 (70–100)	90 (70–100)

Peripheral	32 (49.2)	27 (50)	15 (48.4)	12 (52.2)	6 (50.0)	21 (50.0)
Central	33 (50.8)	27 (50)	16 (51.6)	11 (47.8)	6 (50.0)	21 (50.0)

MGMT promoter methylated	14 (21.5)	14 (25.9)	7 (22.6)	7 (30.4)	3 (25.0)	11 (26.2)
MGMT promoter not methylated	17 (26.2)	14 (25.9)	11 (35.5)	3 (13.0)	3 (25.0)	11 (26.2)
MGMT not determined	34 (52.3)	26 (48.2)	13 (41.9)	13 (56.5)	6 (50.0)	20 (47.6)

Surgical resection	56 (86.2)	46 (85.2)	27 (87.1)	19 (82.6)	9 (75.0)	22 (52.4)
Gross total resection	26 (40.0)	20 (37.0)	10 (32.2)	10 (43.5)	3 (25.0)	11 (26.2)
Subtotal resection	30 (46.2)	26 (48.1)	17 (54.8)	9 (39.1)	6 (50.0)	11 (26.2)
Biopsy	8 (12.3)	7 (13.0)	4 (12.9)	3 (13.0)	3 (25.0)	20 (47.6)
n.d.	1 (1.5)	1 (1.9)	0 (0)	1 (4.3)	0 (0)	1 (2.4)

Volume of PTV in mL (range)	342.0 (117.4–674.7)	280.6 (117.4–674.7)	377 (177.8–674.74)	266 (117.4–520.8)	401.3 (265.2–674.7)	324.3 (117.4–560.6)
Median total dose in Gy (range)	60.0 (40.05–68)	60.0 (59.4–68)	60.0 (59.4–60)	60.0 (59.4–68)	60.0 (59.4–60)	60.0 (59.4–68)
Median single dose in Gy (range)	2.0 (1.8–2.67)	2.0 (1.8–2.0)	2.0 (1.8–2.0)	2.0 (1.8–2.0)	2.0 (1.8–2.0)	2.0 (1.8–2.0)

Gy: gray; MGMT: O-6-methylguanine methyltransferase; IL: Ipsilateral; CL: contralateral; SVZ: subventricular zone; PTV: planning target volume; n.d.: not determined.

**Table 2:** Progression-free and overall survival in regard of dosimetry in glioblastoma patients with conventional fractionated radiotherapy

Cofactors	Number of patients, ≥40 Gy/<40 Gy	Median PFS, ≥40 Gy	Median PFS, <40 Gy	*p* value	Median OS, ≥40 Gy	Median OS, <40 Gy	*p* value
Mean IL ventricle dose	30/24	9.0 (6.3–11.7)	5.1 (3.5–6.8)	0.11	21.6 (18.5–24.7)	18. (11.2–24.8)	0.15
Mean CL ventricle dose	7/47	10.1 (4.1–16.1)	7.2 (5.0–9.4)	0.26	21.6 (12.6–30.6)	21.2 (16.0–26.5)	0.65
Mean IL SVZ dose	31/23	**8.5 (6.3–10.2)**	**5.2 (3.1–7.3)**	0**.01**	21.3 (17.5–25.2)	18.0 (11.4–24.6)	0.19
Mean CL SVZ dose (≥30 Gy versus <30 Gy)	12/42	**10.1 (8.9–11.3)**	**6.8 (4.8–9.0)**	0**.03**	21.6 (12.2–31.0)	21.2 (16.4–26.1)	0.29
Mean IL DG dose	22/32	7.3 (5.4–9.2)	7.8 (5.0–10.5)	0.22	20.8 (12.5–29.1)	21.3 (15.6–27.1)	0.49
Mean CL DG dose (≥30 Gy versus <30 Gy)	4/50	9.4 (0.28–18.6)	7.3 (6.3–8.3)	0.84	15.4 (−5.96–36.8)	21.3 (16.7–25.8)	0.85

CI: confidence interval; HR: hazard ratio; PFS: progression-free survival; OS: overall survival; IL: ipsilateral; CL: contralateral; SVZ: subventricular zone; Gy: gray.

**Table 3:** Univariate proportional-hazards regression analysis of cofactors on progression-free survival in glioblastoma patients with conventionally fractionated radiotherapy.

Cofactors	HR	95% CI	*p* value
Karnofsky performance status > 80	1.16	0.68–1.96	0.59
Peripheral versus central	0.85	0.51–1.39	0.51
MGMT promoter methylation	0.93	0.68–1.34	0.60
Biopsy versus surgical resection	0.48	0.22–1.03	0.06
Gross total resection versus subtotal resection	0.70	0.41–1.40	0.38
Temozolomide therapy	0.60	0.32–1.09	0.09
Mean IL ventricle dose ≥ 40 Gy	**0.56**	0.32–0.98	**0.043**
Mean CL ventricle dose ≥ 40 Gy	0.61	0.26–1.44	0.26
Mean IL SVZ dose ≥ 40 Gy	**0.40**	0.24–0.78	**0.002**
Mean CL SVZ dose ≥ 30 Gy	**0.44**	0.21–0.92	**0.030**
Mean IL DG dose ≥ 40 Gy	1.42	0.81–2.51	0.22
Mean CL DG dose ≥ 30 Gy	0.86	0.31–2.40	0.77

CI: confidence interval; HR: hazard ratio; IL: ipsilateral; CL: contralateral; SVZ: subventricular zone; DG: dentate gyrus; Gy: gray; MGMT: O-6-methylguanine methyltransferase; RT: radiation therapy.

**Table 4:** Univariate proportional-hazards regression analysis of cofactors on overall survival in glioblastoma patients with conventionally fractionated radiotherapy.

Cofactors	HR	95% CI	*p* value
Karnofsky performance status > 80	1.01	0.61–2.04	0.73
Peripheral versus central	1.05	0.59–1.85	0.88
MGMT promoter methylation	0.93	0.67–1.29	0.66
Biopsy versus surgical resection	0.63	0.30–1.51	0.29
Gross total resection versus subtotal resection	0.97	0.52–1.80	0.92
Temozolomide therapy	**0.49**	0.27–0.90	**0.02**
Mean IL ventricle dose ≥ 40 Gy	0.64	0.34–1.21	0.17
Mean CL ventricle dose ≥ 40 Gy	0.80	0.31–2.05	0.65
Mean IL SVZ dose ≥ 40 Gy	0.65	0.34–1.24	0.10
Mean CL SVZ dose ≥ 30 Gy	1.53	0.36–6.43	0.56
Mean IL DG dose ≥ 40 Gy	1.24	0.61–2.32	0.50
Mean CL DG dose ≥ 30 Gy	1.21	0.16–3.09	0.85

CI: confidence interval; HR: hazard ratio; IL: ipsilateral; CL: contralateral; SVZ: subventricular zone; DG: dentate gyrus; Gy: gray; MGMT: O-6-methylguanine methyltransferase; RT: radiation therapy.

**Table 5:** Multivariate proportional-hazards regression analysis of cofactors on progression-free survival in glioblastoma patients with conventionally fractionated radiotherapy.

Cofactors	HR	95% CI	*p* value
Karnofsky performance status > 80	0.83	0.45–1.53	0.55
Peripheral versus central	0.52	0.26–1.03	0.27
Biopsy versus surgical resection	0.72	0.32–1.60	0.42
Temozolomide therapy	0.78	0.40–1.54	0.47
Mean IL SVZ dose ≥ 40 Gy	0.52	0.26–1.03	0.06
Mean CL SVZ dose ≥ 30 Gy	**0.45**	0.20–0.98	**0.04**

CI: confidence interval; HR: hazard ratio; IL: ipsilateral; CL: contralateral; SVZ: subventricular zone; Gy: gray.
